# The antibody-peptide fusion protein, AT-02, is an effective opsonin with pan-amyloid reactivity

**DOI:** 10.1038/s44386-025-00015-4

**Published:** 2025-08-04

**Authors:** J. S. Wall, A. D. Williams, M. Balachandran, T. J. Hancock, T. Richey, A. C. Stuckey, S. Macy, C. Wooliver, E. B. Martin, J. W. Jackson, R. E. Heidel, S. Guthrie, M. L. Klein, N. Angell, S. J. Kennel, J. S. Foster

**Affiliations:** 1 Department of Medicine, UTHSC College of Medicine Knoxville, Knoxville, TN USA; 2 Department of Surgery, UTHSC College of Medicine Knoxville, Knoxville, TN USA; 3 Attralus Inc, San Francisco, CA USA

**Keywords:** Antibody therapy, Pharmaceutics

## Abstract

In systemic amyloidosis, the progressive accumulation of amyloid fibrils along with extracellular matrix components causes organ dysfunction, reduced quality of life, and is often fatal. Currently, twenty different proteins have been identified in systemic amyloid fibrils. Therapeutic elimination of tissue amyloid may improve organ function and patient outcomes. Amyloid-reactive immunoglobulins capable of stimulating cell-mediated clearance of amyloid deposits represent promising therapeutic strategies. AT-02 (zamubafusp alfa) is an immunoglobulin-peptide fusion protein comprising a humanized IgG1 and a pan-amyloid reactive peptide, p5R. AT-02 binds two ubiquitous components of all amyloids, namely amyloid fibrils and highly sulfated heparan sulfate glycans. This fusion protein exhibits specific reactivity with many amyloid types, obviating the need for individualized immunoglobulins for therapy. We demonstrate that AT-02 is an effective opsonin for amyloid by enhancing phagocytosis of amyloid extracts and inducing complement fixation. These data suggest that pan-amyloid binding reagents may provide novel treatment options for amyloid removal.

## Introduction

Amyloid is a complex pathology comprising protease-resistant amyloid fibrils, extracellular matrix components, including heparan sulfate proteoglycans, and serum proteins. In patients with systemic amyloidosis, extracellular amyloid can deposit in any organ or tissue, with cardiac and renal accumulation being the leading causes of mortality. The most common types of systemic amyloidosis result from the aggregation and deposition of wild-type or variant transthyretin (ATTRwt or ATTRv) or of monoclonal immunoglobulin lights chains (AL). ATTR amyloidosis is now recognized as a major cause of heart failure, with more than 150,000 cases in the United States^1^. The prevalence of AL amyloidosis in the US has increased to ~50 per million people^2,3^. Approximately 16 other proteins have been identified in pathologic amyloid fibrils in patients with systemic amyloidosis^4^; however, these types are currently considered extremely rare. The continuing rise in systemic amyloid diagnoses can be attributed to increased disease awareness related to the approval of new therapies and wider adoption of non-invasive diagnostic tools^5,6^. Even though amyloid fibrils are composed of misfolded, non-native proteins and deposit in the extracellular space where they are readily accessible to immune recognition, amyloid does not commonly inspire innate or humoral immunological responses. Consequently, in the absence of effective therapeutic intervention, amyloid can progressively accumulate with coincident worsening of organ function and quality of life.

Reducing the production of the amyloidogenic precursor protein, or lowering the serum concentration by stabilizing the native state, are effective therapeutic strategies for slowing the progression of amyloid deposition^7^. Use of approved or prospective therapeutics that achieve these goals has significantly impacted the overall survival of patients with ATTR and AL-associated amyloidosis^8–10^. In a small subset of patients with AL amyloidosis, a sustained and complete hematologic remission (normalization of the involved serum light chain isotype) in response to therapy may result in a reduction in tissue amyloid and a positive organ response^11,12^. Significant regression of ATTR amyloid is rarely observed, but case reports have described regression of splenic or cardiac ATTR amyloid either in response to therapy^13,14^ or in patients who naturally develop ATTR amyloid reactive immunoglobulin^15^. This latter clinical observation suggests that amyloid-reactive antibodies can enable autogenous clearance of cardiac amyloid and restoration of organ function.

**Fig. 1:**
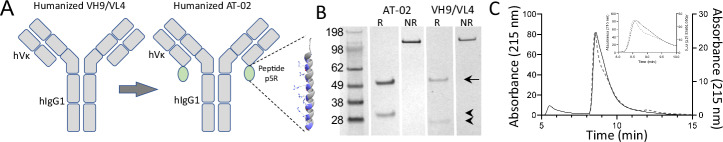
Structure and characterization of the human IgGκ1-peptide fusion AT-02. **A** Humanized IgG1κ clone VH9/VL4 was generated to which the pan-amyloid reactive peptide p5R was genetically incorporated at the C-terminal of the light chain. **B** Reducing (R) non-reduced (NR) SDS-PAGE of AT-02 and VH9/VL4 showing increased mass of the light chain (arrowhead). Molecular weight standards are shown for comparison (kDa). See Supplementary Data for uncropped gel. **C** Size-exclusion HPLC of AT-02 (dashed) and VH9/VL4 (solid) showing increased molecular weight of AT-02.

Therapeutic removal of tissue amyloid is a major clinical goal. Amyloid reactive monoclonal antibodies, specific for AL or ATTR, are being clinically evaluated as opsonins to induce cell-mediated clearance of amyloid and/or sequester oligomeric misfolded proteins that may be cardiotoxic^16^. These reagents bind cryptic epitopes (cryptotopes) exposed on monomeric TTR^17^ or misfolded light chain proteins^18–20^ and thereby avoid binding to native tetrameric TTR, immunoglobulins, and natively folded free light chains. Building on our usage of pan-amyloid peptide reagents for imaging systemic amyloid both in animal models^21^, and more recently in clinical studies with patients^22,23^ we have used polybasic pattern-recognition peptides that bind hypersulfated heparan sulfate and amyloid fibrils^21,24–27^ to generate pan-amyloid reactive opsonins^28,29^. Herein, we characterize the reactivity and bioactivity of AT-02 (zamubafusp alfa), a novel humanized amyloid binding IgG1κ-peptide fusion protein (Fig. 1A). AT-02 binds both amyloid fibrils and exhibits highly selective reactivity with heparan sulfate glycans via electrostatic interactions. The reagent reacts specifically with many types of amyloid deposits, including those in a mouse model of serum amyloid protein A (AA) amyloidosis where it rapidly binds and remains bound for over seven days. AT-02 serves as an effective opsonin when bound to amyloid, thereby enhancing phagocytosis of amyloid extracts and fixing complement. These data demonstrating pan-amyloid activity have motivated the evaluation of AT-02 in patients with systemic amyloidosis (NCT05951049 and NCT05521022).

## Results

### AT-02 (zamubafusp alfa) is a humanized immunoglobulin-peptide fusion

The fusion of the amyloid-reactive peptide to the IgG light chain of AT-02 increased the molecular mass and resulted in a shift in the mobility relative to the light chain band of the VH9/VL4 IgG1 seen by SDS-PAGE under reducing conditions (Fig. 1B, arrowheads). The unmodified heavy chain bands of AT-02 and VH9/VL4 have identical molecular masses (Fig. 1B, arrow). The additional amino acids on the C-terminal end of AT-02 light chain increase the molecular mass by ~3530 Da. The size exclusion chromatograms of AT-02 and VH9-VL4 were consistent with small increase in hydrodynamic radius (Fig. 1C). The theoretical molecular mass of the partially reduced light chain-peptide fusion (27,574.9) and deglycosylated heavy chain (47,818.1) correlated well with the observed values of 27,572.5 and 47,814.5, respectively (Supplementary Fig. 1). These data were within a mass accuracy of 90 ppm and varied due to N-terminal glutamine modification to pyro-glutamic acid with and without the loss of C-terminal lysine (-K) of the heavy chain (Supplementary Fig. 1).

### AT-02 binds charged glycosaminoglycans and amyloid fibrils

**Fig. 2:**
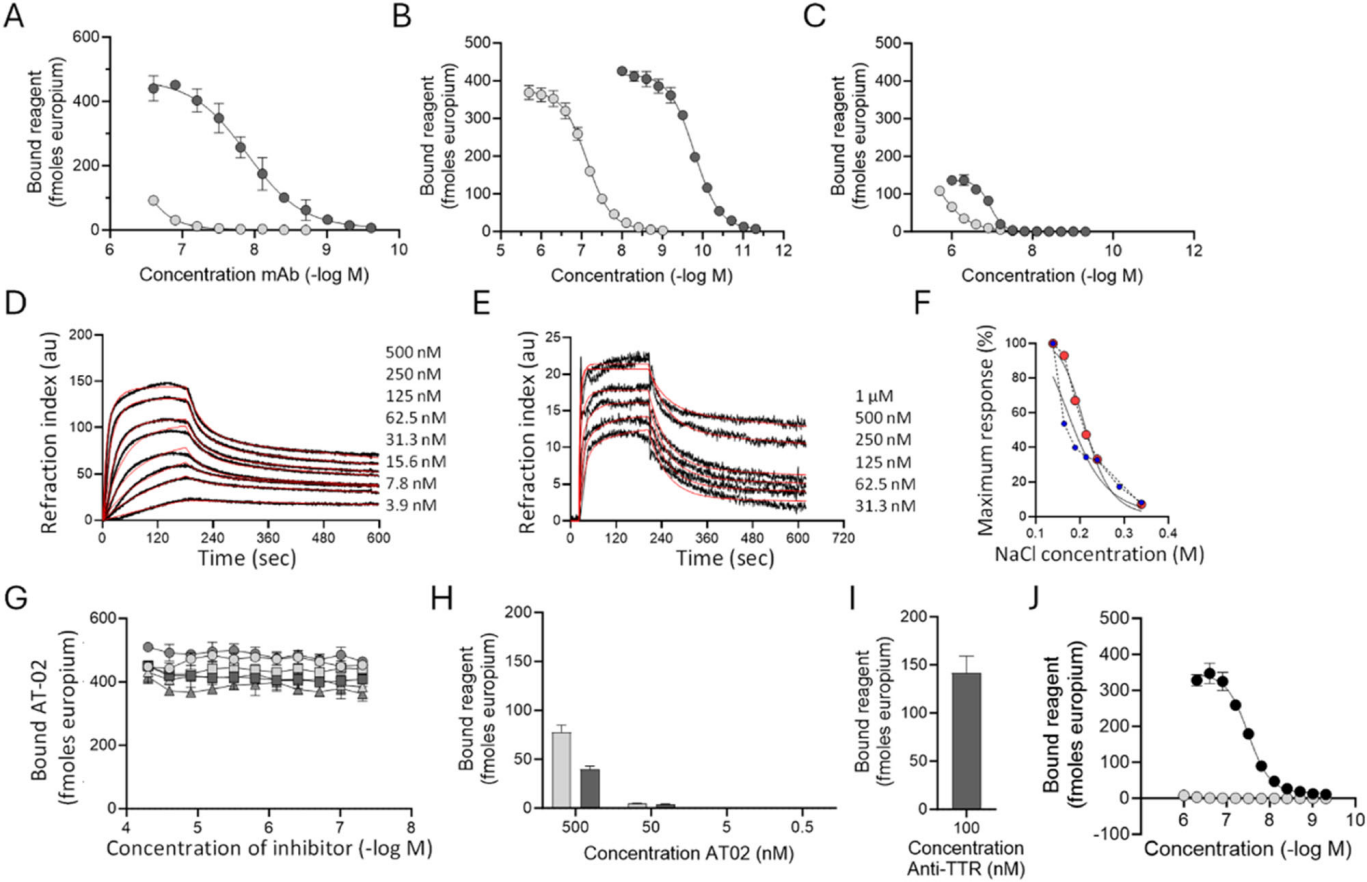
Incorporation of peptide p5R enhances binding of AT-02 to amyloid-like fibrils and hypersulfated glycosaminoglycans. **A** Binding of AT-02 (dark) and VH9/VL4 (light) to heparin coated plates. **B** Binding of AT-02 (dark) and VH9/VL4 (light) to synthetic light chain rVλ6WIL amyloid-like fibrils. **C** Binding of AT-02 (dark) and VH9/VL4 (light) to non-fibrillar rVλ6WIL proteins. Surface plasmon resonance (SPR) of AT-02 binding to rVλ6WIL amyloid-like fibrils (**D**) and heparin (**E**). **F** Inhibition of AT-02 (500 nM) binding to rVλ6WIL synthetic fibrils (triangles) and heparin (circle) assessed by SPR in the presence of increasing NaCl. **G** Binding of AT-02 (1 nM) to rVλ6WIL fibrils in the presence of increasing concertation of precursor proteins: TTRwt (light square); TTRv soluble aggregates (dark square); λ6 light chain (dark circle); λ2 light chain (light circle); κ4 light chain (dark triangle); κ1 light chain (light triangle). **H** Binding of AT-02 to 100 nM TTRv soluble aggregates (light) and TTRwt (dark) captured in an ELISA assay. **I** Positive control capture of TTRwt using anti-TTR mAb. **J** Binding of AT-02 (dark) and control hIgG1 (light) to TTRv soluble aggregates (0.83 uM stock) coated onto the wells of a microplate.

Peptide p5R was developed to target hypersulfated heparan sulfate (HS), which is ubiquitously associated with all types of amyloid deposits. Heparin is the most sulfated glycosaminoglycan and can serve as a surrogate for the hypersulfated domains of HS. AT-02 bound to surface immobilized heparin with high potency (EC_50_ = 12.5 nM), whereas no reactivity of VH9/VL4 was observed at concentrations up to 500 nM (Fig. 2A). In addition to heparin, AT-02 bound with high potency to synthetic rVλ6WIL fibrils (EC_50_ = 0.16 nM) (Fig. 2B) while the VH9/VL4 IgG1 bound rVλ6WIL fibrils with much reduced potency (EC_50_ = 74.5 nM) (Fig. 2B). Neither AT-02 nor VH9/VL4 exhibited significant binding to the plate-immobilized rVλ6WIL monomer by ELISA (Fig. 2C).

The kinetics of AT-02 binding to rVλ6WIL fibrils covalently immobilized on a surface plasmon resonance (SPR) chip were assessed over the concentration range of 3.9 nM to 500 nM (Fig. 2D). The binding data were best approximated by a two-site binding model with high affinity (*k*a = 2.6 × 10^5^ M*s^−1^; *k*d = 9.9 × 10^−4^ sec^−1^ and KD = 3.7 nM) and low affinity (*k*a = 5.6 × 10^4^ M*s^−1^; *k*d = 2.3 × 10^−2^ sec^−1^ and KD = 406 nM) interactions and with a Chi^2^ = 4.18 (Fig. 2D). No binding of VH9/VL4 to rVλ6WIL fibrils was detected under these conditions. Using a low-density heparin SPR chip, AT-02 similarly exhibited high affinity (*k*a = 3.1 × 10^5^ M*s^−1^; *k*d = 3.6 × 10^−4^ sec^−1^ and KD = 1.2 nM) and low affinity (*k*a = 1.7 × 10^6^ M*s^−1^; *k*d = 2.1 × 10^−2^ sec^−1^ and KD = 12.2 nM) interactions, with a Chi^2^ = 0.67 (Fig. 2E). VH9/VL4 did not bind heparin in this assay. A two-site binding model may best describe the interaction of AT-02 to immobilized rVλ6WIL fibrils because there are two p5R peptides per IgG allowing mono- and bivalent binding, and the reactivity of the F(ab)_2_ may also contribute. Electrostatic interactions are proposed to mediate the binding of AT-02 to amyloid fibrils and hypersulfated HS. Therefore, the effect of increasing sodium chloride (NaCl) on binding was assessed using SPR. Increasing NaCl concentration in the reaction solution from 130 mM to 350 mM reduced the binding of AT-02 to both fibrils (IC_50_ = 215 nM NaCl) and heparin (IC_50_ = 187 nM NaCl) on the SPR chip (Fig. 2F).

### AT-02 does not bind free immunoglobulin light chain or non-fibrillar transthyretin

The binding of AT-02 at 1 nM (~EC_90_ value) to immobilized rVλ6WIL fibrils was assessed in the presence of human Ig κ and λ light chains, and TTR in solution. Over a concentration range of 50 nM to 50 μM, none of the competitor light chains, TTR tetramer, or soluble thioflavin T-negative TTR aggregates inhibited the binding of AT-02 to the fibrils (Fig. 2G). To further assess the reactivity of AT-02 with native TTR tetramer and soluble TTRv aggregates a capture ELISA was performed. No binding of AT-02 was observed to TTR tetramer nor to TTRv aggregates, at concentrations of 50 nM and lower (Fig. 2H). At 500 nM AT-02, modest binding to the captured TTR species was observed (3125-fold higher than the EC_50_ for rVλ6WIL fibril binding). A TTR-reactive IgG control (at 100 nM) had significantly higher binding to TTR tetramers in this assay format (Fig. 2I). When the soluble TTRv aggregates were immobilized directly onto the wells of the microplate, the binding of AT-02 in the ELISA assay became evident (EC_50_ = 31.6 nM), whereas no binding of a non-specific hIgG1 was observed (Fig. 2J). Surface adsorbed TTRv aggregates may present amyloid-like neo-epitopes due to surface-mediated misfolding and potentially increasing local concentrations, which allowed the AT-02 interactions.

### Reactivity with highly charged heparan sulfate

**Fig. 3:**
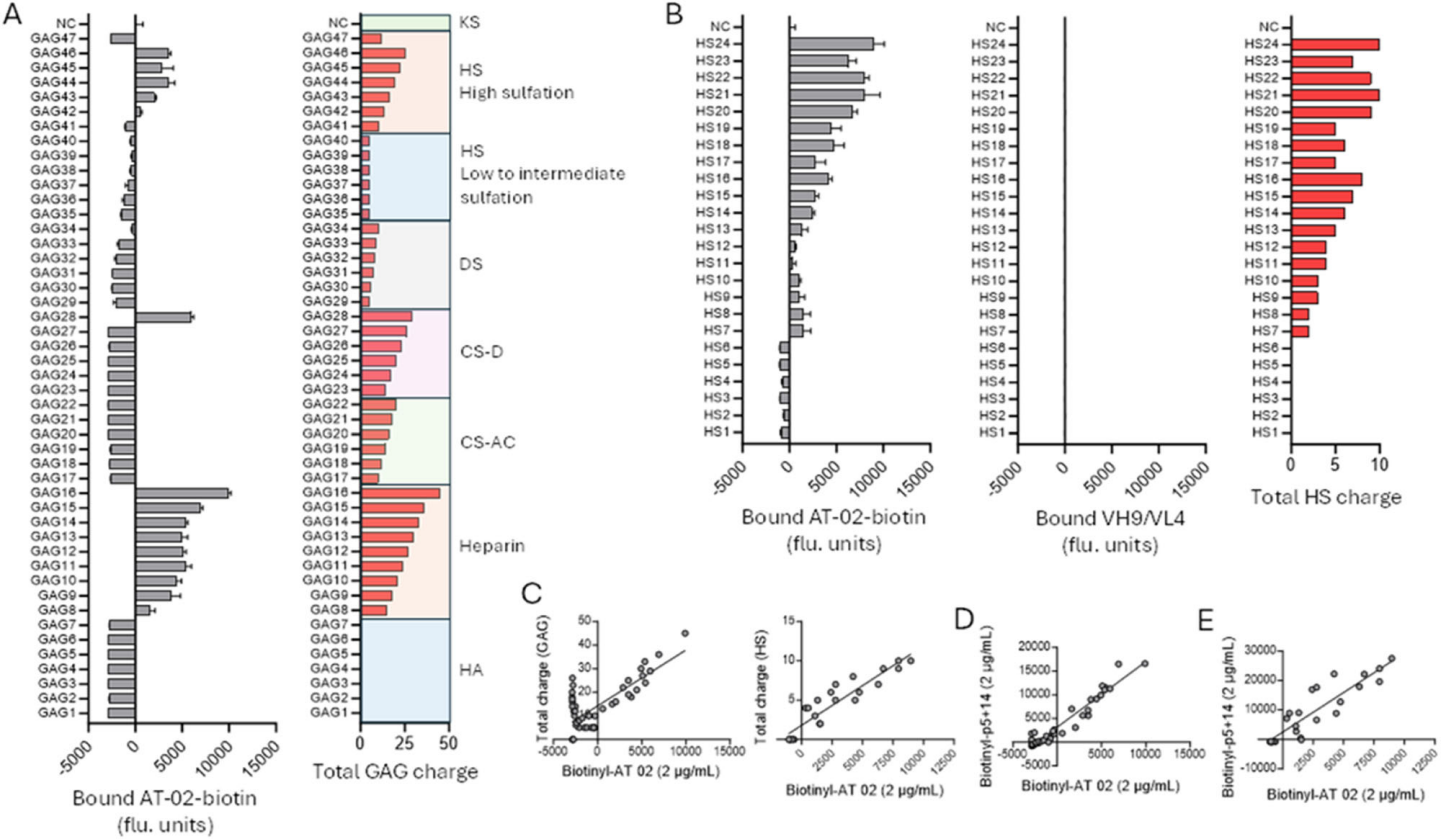
AT-02 specifically binds hypersulfated and heparan sulfate glycosaminoglycans. **A** Binding of biotinylated AT-02 to a glycosaminoglycan array. **B** Binding of biotinylated AT-02 or VH9/VL4 to a heparan sulfate array. **C** Correlation analysis of biotinylated AT-02 binding and the total charge on glycosaminoglycan (left) or heparan sulfates (right). **D** Correlation analysis of the binding of biotinylated AT-02 and AT-01 to glycosaminoglycan or heparan sulfate charge (**E**). Correlations were performed using two-tailed Pearson analysis (*r*_P_) with α = 0.05.

Biotinylated AT-02 (2 μg/mL) exhibited highly selective binding to specific glycans in the glycosaminoglycan array. No reactivity was observed with the hyaluronic acid (HA), chondroitin sulfate AC (CS-AC), dermatan sulfate (DS), or keratan sulfate (KS) glycans (Fig. 3A). Binding was observed for members of the heparin and heparan sulfate (HS) family and a single chondroitin sulfate D glycan. In the case of heparin, the extent of binding increased proportional to the number of disaccharides and the total charge on glycan, with greatest binding observed to the most negatively charged heparin (GAG16) (Fig. 3A). A similar charge-dependent pattern of binding was observed for the HS family. Only one highly sulfated marine CS-D glycan with nine disaccharide repeats was recognized by biotinylated AT-02 (Fig. 3A).

The relationship between biotinylated AT-02 binding and the charge and structure of HS chains was further explored using a specific HS array (Fig. 3B, Supplementary Fig. 3). Biotinylated AT-02 did not bind uncharged HS (HS1-HS6). Introduction of N-sulfation (HS7-HS13) resulted in modest binding; however, when the glycans contained 6S and 2S sulfations, the binding of biotinylated AT-02 increased dramatically and proportional to the net negative charge (Fig. 3B). Iduronic acid (IdoA) substitutions for glucuronic acid (GlcA) in the absence of 6S sulfation in HS17-HS19 modestly reduced biotinylated AT-02 binding; however, addition of 6S sulfation resulted in enhanced reactivity (HS20-HS23). In general, highest binding of AT-02 was observed when HS glycans contained saccharides with, either 6S and NS, or 3S and NS sulfations (Fig. 3B).

There was a strong significant correlation between biotinylated AT-02 binding and the total charge on the glycosaminoglycans (*r*_P_ = 0.76, *p* < 0.0001) or HS (*r*_P_ = 0.93, *p* < 0.0001) (Fig. 3C). The selective binding of AT-02 to the glycosaminoglycan and HS species was also compared to that of biotinylated peptide p5+14, a lysine-based peptide that is being used to detect systemic amyloid in patients using PET/CT imaging (iodine ^124^I evuzamitide). The binding intensity of biotinylated AT-02 to the glycosaminoglycan (*r*_P_ = 0.97, *p* < 0.0001) and HS (*r*_P_ = 0.90, *p* < 0.0001) arrays correlated significantly with that of biotinylated p5+14 (Fig. 3D, E).

### AT-02 specifically binds many amyloid types with high potency

**Fig. 4:**
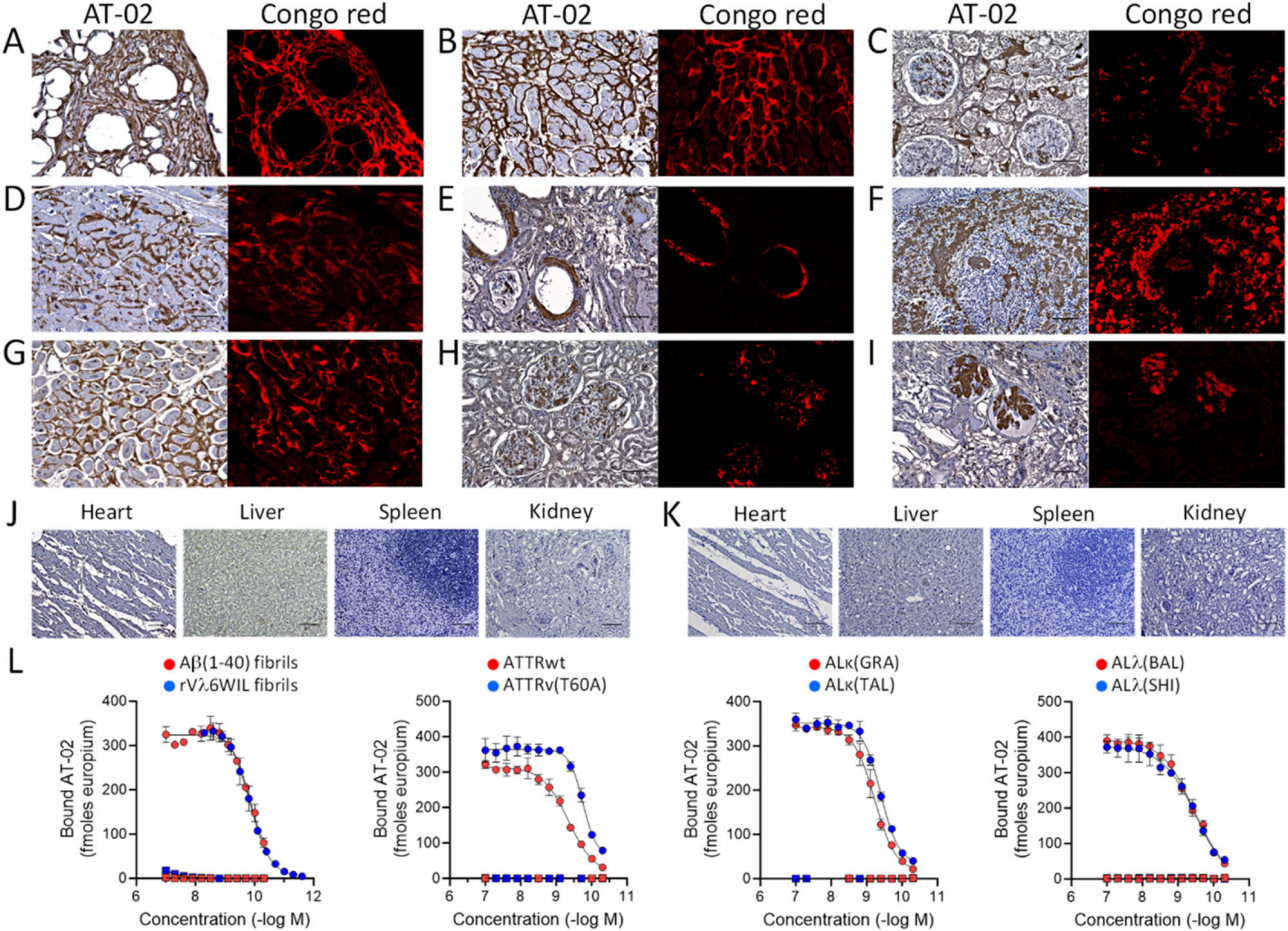
AT-02 binds diverse types of human amyloid. Immunoreactivity of biotinylated AT-02 with Congo red-positive: **A** ALκ(TAL) heart; **B** ALλ(BAB) heart; **C** ALECT2 kidney; **D** ATTRv(SNO) heart; **E** ALλ(JON) kidney; **F** ALECT2 spleen (bar = 50 µm); **G** ATTRv(KEN) heart; **H** AL(HUN) kidney and; **I** ALλ(JON) kidney. **J** Immunoreactivity of biotinylated AT-02 and **K** control hIgG1 with amyloid-free tissues, bar = 50 µm. Bar = 100 µm unless otherwise indicated. **L** Binding of AT-02 (circles) and control hIgG1 (squares) to amyloid-like fibrils and human amyloid extracts. The mean ± SD of *n* = 3 or 4 replicates were plotted and the data analyzed using a four-parameter logistic regression where the x-axis is log values.

The specificity and reactivity of AT-02 for amyloid of different types was evaluated immunohistochemically using formalin-fixed amyloid-laden tissues and amyloid extracted from patient organs. Biotinylated AT-02 specifically bound cardiac amyloid deposits composed of Igκ (Fig. 4A), Igλ (Fig. 4B) and ATTRv (Fig. 4D, G). Biotinylated AT-02 binding to perivascular (Fig. 4E) and glomerular AL amyloid deposits was observed (Fig. 4C, H, I) as was binding to interstitial renal ALECT2 and perifollicular splenic ALECT2 amyloid (Fig. 4F). No binding of biotinylated AT-02 or the non-specific biotinylated hIgG1 to amyloid-free tissue samples from the heart, liver, spleen, or kidney was observed (Fig. 4J, K).

The potency of AT-02 binding to diverse amyloid-like fibrils and human amyloid extracts was assessed and compared to a human IgG1 negative control using an ELISA (Fig. 4L). The amyloid extracts contain, in addition to fibrils, all the components of the amyloid pathology observed in tissues. The binding to synthetic amyloid-like fibrils was potent with subnanomolar EC_50_ estimates for rVλ6WIL (EC_50_ = 0.16 nM) and Aβ(1-40) (EC_50_ = 0.15 nM) fibrils. Similarly potent binding was observed for human amyloid extracts, including ATTRv (EC_50_ = 0.45 nM) and ATTRwt (EC_50_ = 0.18 nM) cardiac amyloid as well as ALκ (ALκ[GRA] EC_50_ = 0.60 nM and ALκ[TAL] EC_50_ = 0.40 nM) and ALλ (ALλ[BAL] EC_50_ = 0.36 nM and ALλ[SHI] EC_50_ = 0.32 nM) amyloid (Fig. 4L). No binding of the negative control human IgG1 was observed for any fibril or amyloid substrate up to concentrations of 100 nM.

### AT-02 enhances the phagocytosis of AL and ATTR amyloid via FcγR-mediated interactions and fixes complement in the presence of human plasma

**Fig. 5:**
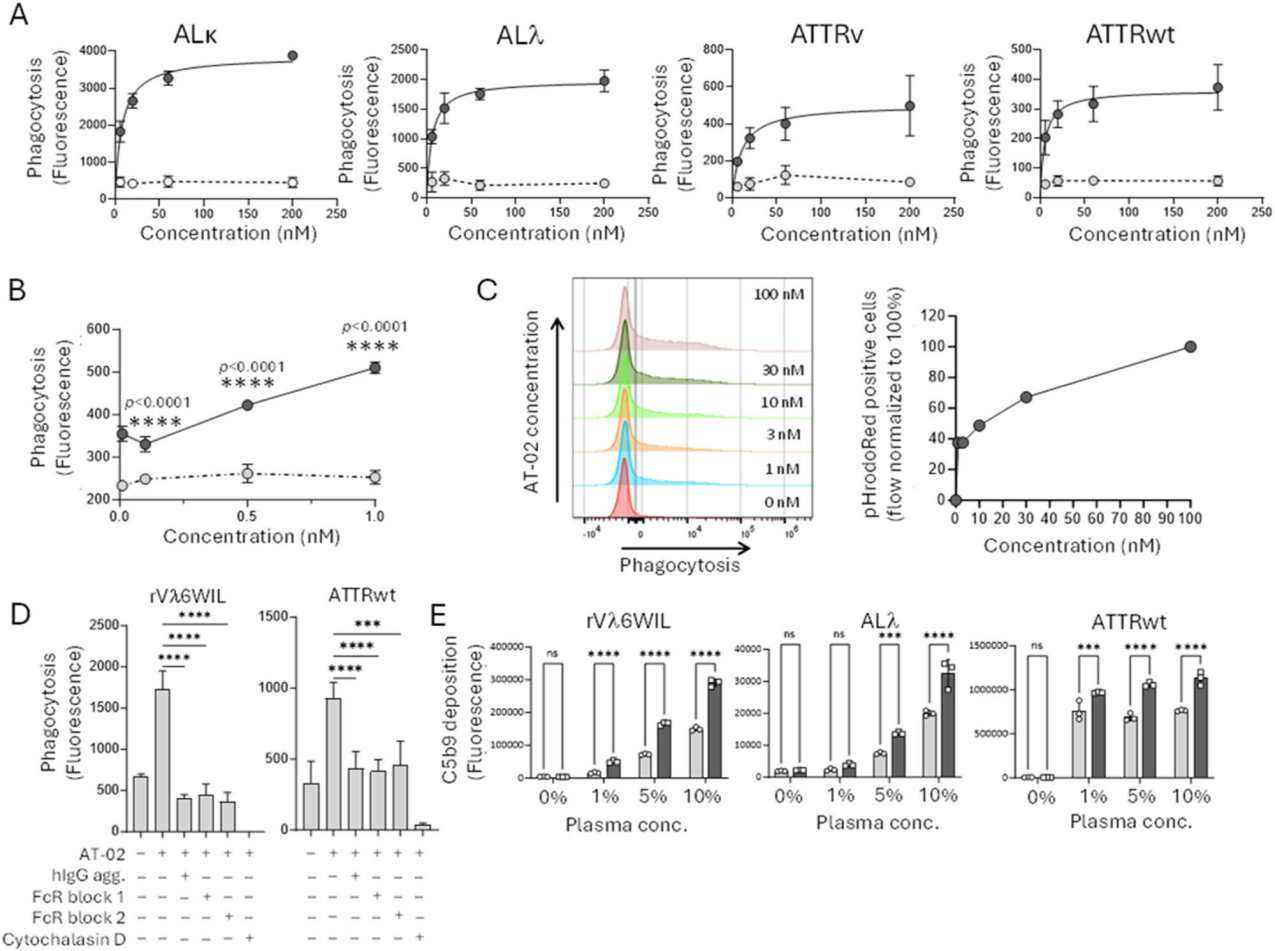
AT-02 binding induces phagocytosis of amyloid by human macrophages mediated through FcR interaction and induces complement activation. **A** Phagocytosis of human AL and ATTR amyloid extracts by PMA-activated THP-1 macrophages in the presence of AT-02 (black) or hIgG1 (white) after 1 h of incubation. **B** Phagocytosis of ATTRwt amyloid extract by PMA-activated THP-1 macrophages in the presence of sub-nanomolar concentration of AT-02 (black) or control hIgG1 (white). **C** Phagocytosis of surface-adsorbed ATTRwt amyloid extract after 3 h of incubation by THP-1 monocytes induced by increasing concentrations of AT-02 monitored by flow cytometry and fluorescence microscopy. **D** Phagocytosis of rVλ6WIIL fibrils and ATTRwt by PMA-activated THP-1 inhibited by FcR inhibitors and competitors. **E** Complement activation and formation of C5b9 complex in the presence of AT-02 and human plasma. In A-D amyloid extracts and synthetic rVλ6WIL fibrils were labeled with NHS-pHrodo Red. Data were analyzed using a 2-way ANOVA, α = 0.05 with Šidák’s correction for multiple comparisons (**B**, **E**) or a one-way ANOVA, α = 0.05, using Dunnett’s correction for multiple comparisons (**D**).

**Fig. 6:**
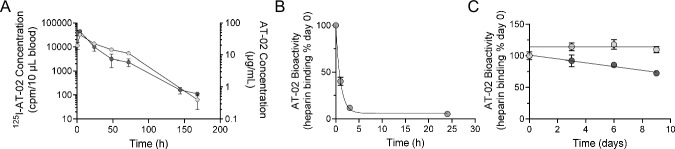
AT-02 has a short half-life in mice and is susceptible to degradation by mouse serum but not human serum. **A** PK profiles of ^125^I-AT-02 (dark) and AT-02 (light) following IV bolus injection of ~5 mg/Kg. The bioactivity of AT-02 incubated in mouse serum, measured by heparin binding, decreased rapidly (**B**) but was more stable under the same experimental conditions in human serum (**C**, dark). No loss of bioactivity was seen over 9 days in PBS (**C**, light). Data were analyzed using a 2-way ANOVA, α = 0.05 with Šidák’s correction for multiple comparisons (A).

Antibody-mediated clearance of tissue amyloid is thought to involve opsonization followed by induction of cell-mediated phagocytosis and destruction of the amyloid. Effective clearance of amyloid may require the recruitment of peripheral blood mononuclear cells, in response to complement activation. The AT-02-mediated uptake of AL and ATTR amyloid was measured using human extracts labeled with the pH-sensitive fluorophore pHrodo Red. The phagocytosis of labeled human ALκ, ALλ, ATTRv, or ATTRwt by PMA-activated human THP-1 macrophages was enhanced in a dose-dependent manner by the addition of AT-02 (Fig. 5A). The increase in fluorescence emission associated with the uptake of the amyloid into the acidified compartments of the cell was greater for the AL amyloid extracts as compared to the ATTR due to the decreased efficiency of pHrodo Red labeling of the ATTR extracts. Under these experimental conditions (1×10^6^ adherent THP1 macrophages and 20 μg of extract), a concentration of 60 nM AT-02 induced maximal phagocytosis. The half maximal concentrations of AT-02 in the phagocytosis assay were estimated to be: ALκ (7.7 nM), ALλ (5.8 nM), ATTRv (10.6 nM), and ATTRwt (5.2 nM). No uptake of amyloid was observed in the presence of a control hIgG1 (Fig. 6A, dashed). The phagocytosis of pHrodo Red-labeled ATTRwt in the presence of AT-02 was significantly enhanced, relative to a control hIgG1, even at concentrations below 1 nM AT-02 (Fig. 5B).

To mimic tissue amyloid, the ability of THP-1 monocytes to phagocytose pHrodo Red-labeled ATTRwt extract (30 μg) when adsorbed to a collagen-coated substrate in the presence of AT-02 was evaluated using flow cytometry. The number of pHrodo Red-positive cells increased coincident with increasing AT-02 (1 nM to 100 nM) with 50% of the maximal cell number observed at 10 nM AT-02 (Fig. 5C). AT-02-mediated phagocytosis of rVλ6WIL fibrils and ATTRwt amyloid extract by THP-1 macrophages was significantly inhibited by FcR blocking agents and heat-aggregated human IgG (hIgG agg.) (Fig. 5D). The microfilament disruptor cytochalasin D was used as a positive control to prevent FcR-mediated and scavenger receptor uptake of amyloid (Fig. 5D).

Incubating rVλ6WIL fibrils, AL, or ATTRwt cardiac extract with increasing concentrations of human plasma and AT-02 induced the formation and deposition of complement C5b9 complex (Fig. 5E). The ATTRwt cardiac extract in the presence of the negative control IgG1 caused C5b9 formation at high levels; however, this was further enhanced by the presence of AT-02.

### Pharmacokinetics and serum stability of AT-02

Pharmacokinetic parameters were assessed in amyloid-free, wild-type mice using both iodine-125-labeled AT-02 with radioactivity measurement, and unlabeled AT-02 measured using a capture ELISA. Both PK profiles appeared monoexponential with no distinct alpha and beta phases but with a prominent delayed Tmax at 4 h post injection (Fig. 6A). The terminal half-life (t_1/2_) calculated using the last 4 timepoints, for both ^125^I-AT-02 and AT-02, was similar, 22.9 h and 24 h, respectively. The *r*^2^ for both datasets was >0.97. The datasets were comparable; however, over the first 2 days (three timepoints), the *t*_1/2_ for ^125^I-AT-02 was faster (11.6 h) as compared to AT-02 (28.4 h). This is likely due to ^125^I-AT-02 dehalogenation occurring at the early time points post-injection.

The bioactivity of AT-02 post mouse or human serum incubation was strikingly different. In mouse serum at 37 °C, heparin reactivity rapidly declined within 24 h (Fig. 6B). In contrast, when evaluated in human serum, AT-02 bioactivity remained stable, decreasing only 28% over 9 days of incubation at 37°C (Fig. 6C).

### AT-02 binds specifically and remains bound to amyloid in mice with severe systemic amyloidosis

**Fig. 7:**
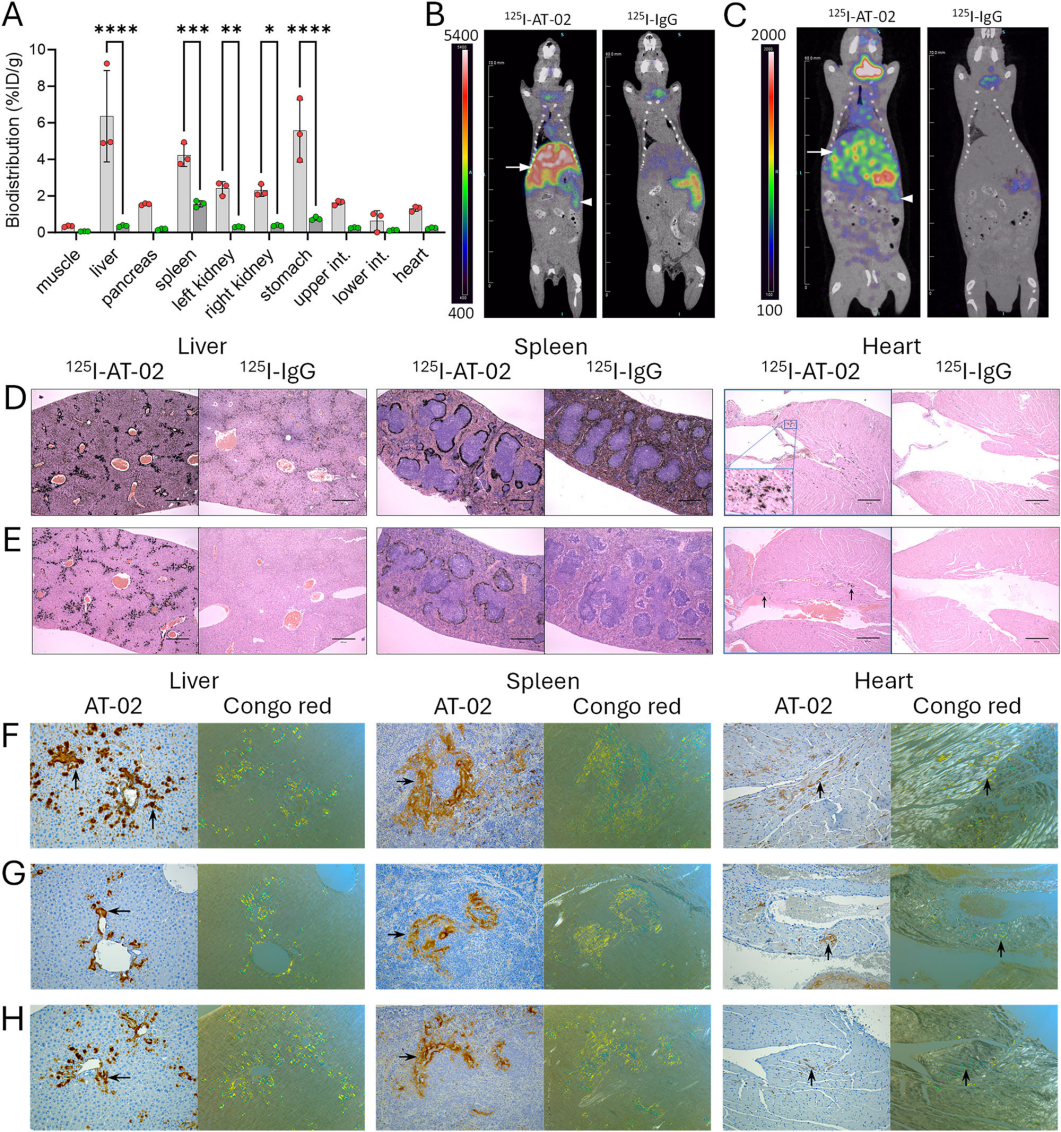
Radiolabeled AT-02 binds tissue amyloid deposits in vivo. **A** Tissue biodistribution of ^125^I-AT-02 in mice with systemic AA amyloidosis (red) compared with ^125^I-hIgG1 (green) at 24 h post injection. Biodistribution of ^125^I-AT-02 and ^125^I-hIgG1 in AA mice at 4 h (**B**) and 24 h (**C**) post injection assessed by small animal SPECT/CT imaging. Binding of ^125^I-AT-02 in AA amyloid deposits in the liver, spleen, and heart was compared to ^125^I-hIgG1 at 4 h (**D**) and 24 h (**E**) post injection using microautoradiography (bar = 500 μm). The AA amyloid-bound lifetime was assessed in the liver, spleen, and heart at 24 h (**F**), 72 h (**G**) and 168 h (**H**) post-injection of AT-02 using immunohistochemistry (20x objective).

Despite the observed instability of AT-02 in mouse serum, at 24 h post IV injection, radiolabeled ^125^I-AT-02 accumulated in all organs of mice with severe systemic serum amyloid protein A (AA) amyloidosis with higher %ID/g than the ^125^I-hIgG1 control (Fig. 7A). When compared to the ^125^I-hIgG1 (IgG) control, the liver (*p* < 0.0001), spleen (*p* = 0.0004), and stomach (*p* < 0.0001) had significantly more ^125^I-AT-02 with approximately 5% injected dose per gram (%ID/g) in each organ. Small animal SPECT/CT imaging confirmed the uptake of ^125^I-AT-02 in the liver (arrow) and spleen (arrowhead) of AA mice at 4 h (Fig. 7B) and 24 h (Fig. 7C) post-injection.

Specific colocalization of ^125^I-AT-02 with hepatic, splenic and cardiac AA amyloid in the mice was assessed using microautoradiography. At 4 h post-injection the deposition of silver grains, indicative of the presence of ^125^I-labeled protein, was seen in the hepatic sinusoids, the perifollicular region of the spleen, and the scant cardiac amyloid deposits seen at the base of the left ventricle (Fig. 7D). At 24 h post-injection, after clearance of the blood pool ^125^I-AT-02 amyloid binding was still evident. In contrast, apart from ^125^I-IgG1 in the splenic red pulp, there was no binding of the ^125^I-IgG1 in the amyloid deposits (Fig. 7E). The density of the silver stain in the microautoradiographs was diminished at 24 h, possibly due to dehalogenation of the ^125^I-AT-02.

The stability of the AT-02-amyloid interaction in the mice was also evaluated immunohistochemically using unlabeled AT-02. Immunostaining of the liver, spleen, and heart harvested at 24 h (Fig. 7F), 72 h (Fig. 7G) and 168 h (Fig. 7H) post-injection of AT-02 (400 μg) showed specific uptake in amyloid within 24 h that persisted for at least 168 h post injection the last timepoint. The distribution of amyloid in the tissues was confirmed by the presence of green-gold birefringence in Congo red-stained consecutive tissue sections.

### AT-02 binds rapidly to human AL extract in mice and enhances phagocytosis and clearance of amyloid in vivo

**Fig. 8:**
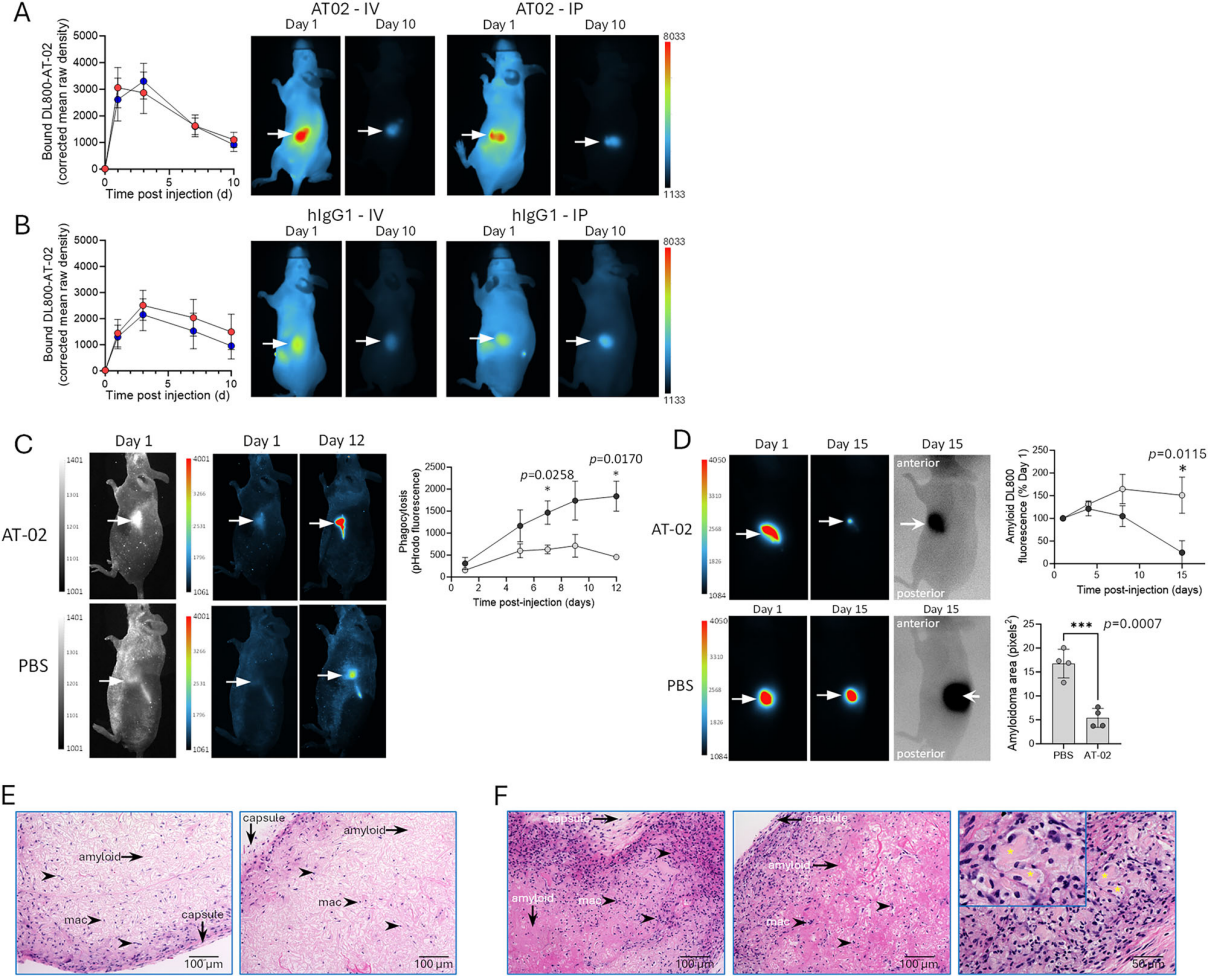
AT-02 rapidly binds subcutaneous human ALλ amyloid in mice and can enhance phagocytosis and clearance. **A** Dylight800-labeled AT-02 (400 μg), when administered either IV (red symbols) or IP (blue symbols), rapidly accumulated in subcutaneous human ALλ amyloidomas and is retained up to 10 days post-injection as evidenced by serial optical imaging. **B** Dylight800-labeled hIgG1 also accumulated, to a lesser degree, at the site of the human AL amyloidoma. **C** AT-02 (400 μg) pretreatment of pHrodo Red-labeled human ALλ amyloid enhanced phagocytosis as compared to control animals evidenced by serial optical imaging and region of interest measurement background-corrected fluorescence mean raw density. **D** AT-02 (400 μg) pretreatment of Dylight800-labeled human ALλ amyloid significantly enhanced clearance of the mass, as compared to control animals, evidenced by serial optical imaging, background-corrected fluorescence mean raw density, and post-mortem measurement of the area of the residual amyloid mass in situ. **E** Histological (hematoxylin and eosin, H&E) evaluation of the residual Dylight800-labeled amyloidoma harvested at 15-day post injection from mice treated with PBS showing the presence of eosinophilic amyloid, macrophages (mac and arrowheads) and the characteristic capsule of the amyloidoma. **F** Residual Dylight800-labeled amyloidoma harvested at 15-day post injection from mice treated with AT-02 showing enhanced macrophage infiltration and amyloid taken up by macrophages (inset). Data were analyzed using a 2-way mixed effects ANOVA, α = 0.05 with Šidák’s correction for multiple comparisons (**C** and **D**) or a Student’s *t*-test, α = 0.05 (**D**).

To evaluate the uptake of AT-02 in human amyloid and assess its ability to enhance in vivo phagocytosis and amyloid clearance, the human AL amyloidoma mouse (NU/NU) model was used. Uptake was evaluated using AT-02 labeled with the near-infrared fluorophore Dylight800 (DL800) administered IV or IP seven days following subcutaneous implantation of 10 mg of human ALλ extract. The uptake of DL800-AT-02, quantified from serial fluorescence images, indicated rapid colocalization within 1-day post-injection, regardless of the route of administration (Fig. 8A). The amyloid-associated fluorescence emission decreased linearly from 3 to 10 days post-injection but remained visible in the optical images. When a non-specific DL800-labeled IgG1 was assessed in the same mouse model, the rate of accumulation at the site of the amyloid was slower, peaking at day 3 and to a lesser degree when compared to the DL800-AT-02 (Fig. 8B).

Phagocytosis was evaluated using human AL amyloid labeled with pHrodo Red. Due to the lack of vasculature in of human AL amyloidomas in mice, the labeled amyloid was pretreated with AT-02 prior to implantation. Phagocytosis of the amyloid in vivo, was significantly enhanced by AT-02 (*p* = 0.0006 for treatment effect) relative to the control, PBS treated amyloid (Fig. 8C). Using a similar experimental design, but with DL800-labeled amyloid extract, AT-02 treatment significantly enhanced clearance of the amyloidoma (*p* = 0.0058 for treatment effect), as measured by the sustained decrease in fluorescence emission from day 3 to day 15 post injection (Fig. 8D). Histological evaluation of the residual amyloidomas excised at day 15 showed a characteristically well-encapsulated amyloidoma with macrophage infiltration which was visibly enhanced in the AT-02-treated animals as compared to the controls (Fig. 8E and F). Eosinophilic amyloid was observed in the macrophages in the AT-02-treated mice (Fig. 8F inset). These data were consistent with a significant reduction in the amyloid lesion, mediated by macrophage uptake in the AT-02-treated mice (Fig. 8).

## Discussion

Amyloid is a complex, devastating pathology that compromises organ function due to progressive amyloid infiltration in the extracellular space. In addition to fibrils composed of misfolded proteins, another major component of amyloid is heparan sulfate (HS) proteoglycans, which provide the etymological foundation of the term amyloid, meaning starch-like, from the Latin ‘*amylum*’. Amyloid-specific HS served as the original target for our identification of pan-amyloid reactive peptides. The κ light chain in AT-02 is comprised of 219 amino acids with a C-terminal extension consisting of a short spacer and a 26 amino acid p5R amyloid binding domain (Fig. 1A). The p5R sequence is based on heptad repeats of Arg-Ala-Gln-Arg-Ala-Gln-Ala with a total of 16 arginine residues on each AT-02 molecule capable of multivalent electrostatic interactions with highly negatively charged components of amyloid deposits. In amyloid, these components are the amyloid fibrils and the highly sulfated HS S-domains, which may themselves contribute to amyloidogenesis^30^.

Our studies have demonstrated that AT-02 is capable of binding amyloid-like fibrils, composed of light chain variable domains (rVλ6WIL) or Aβ(1-40), and heparin, as a surrogate for the HS S-domains (Figs. 2 & 3), with both interactions mediated through the p5R peptide (Fig. 2). In addition, AT-02 bound AL, ATTR, and ALECT2 tissue amyloid by immunohistochemistry as well as AL and ATTRv and ATTRwt amyloid extracts, the latter with sub-nanomolar EC_50_ binding constants (Fig. 4). AT-02 preferentially bound amyloid even in the presence of amyloidogenic monoclonal ALκ or ALλ free light chains, native tetrameric ATTRwt, and non-fibrillar soluble ATTRv aggregates (Fig. 2). AT-02 remained stable in human serum but lost bioactivity in mouse serum; however, AT-02 rapidly and specifically bound murine AA amyloid and human AL amyloid implants in mice and was retained in the AA amyloid for more than 7 days (Figs. 7 & 8). When bound to amyloid, AT-02 induced complement fixation and enhanced phagocytosis of amyloid by human macrophages and monocytes ex vivo (Fig. 5) which, in mice, resulted in rapid macrophage-mediated clearance of human AL amyloid implants (Fig. 8). These data suggest that AT-02 can bind two ubiquitous components of amyloid and, therefore, serves as an effective opsonin for all amyloid types.

In the murine precursor of AT-02 (murine Igp5), as in our peptide imaging agent (^124^I-evuzamitide), a lysine-based peptide, p5, was used as the amyloid binding motif^23,24,28^. In AT-02, this was replaced with peptide p5R, with lysine to arginine substitutions. Relative to lysine-based p5, peptide p5R exhibits greater affinity for immobilized heparin^26^. The in situ formation of an α-helix in the amyloid binding domain is likely important for the interaction with amyloid, and the use of arginine in place of lysine is thought to provide a higher propensity for α-helicity^31^. It has been previously shown that a similar arginine-based peptide could readily adopt an α-helical secondary structure and bound heparin with higher affinity relative to its lysine-substituted analog^32^. The enhanced binding is possibly due to more favorable hydrogen bonding between the heparin and the guanidino side chain or arginine^33^. Thus, AT-02’s affinity for amyloid has improved relative to the murine Igp5 precursor.

Targeting amyloid-associated HS as a therapeutic approach for amyloidosis is not novel^34,35^ because HS is a critical requirement for the development of certain types of amyloid^36,37^. Moreover, amyloid-associated HS is biochemically distinct from that in normal tissues^38,39^ rendering it a druggable target for the treatment of amyloidosis. The selective reactivity of AT-02 for HS and high binding potency is dependent on the total charge as is the amyloid imaging peptide p5+14 (Fig. 3C, D). Peptide p5+14, when radiolabeled (iodine ^124^I evuzamitide), has been used to image cardiac, renal and other abdominothoracic amyloid deposits in patients with diverse types of amyloid by PET/CT imaging^22,23^. Given the relationship between AT-02 and p5 + 14 binding characteristics, the amyloid reactivity of p5+14 observed in patients may predict the in vivo behavior of AT-02. Accumulation of AT-02 in renal amyloid, notably in the renal medulla may be negatively impacted by the high salt concentrations; however, based on preclinical and clinical observations, electrostatic interactions of peptides with cortical and glomerular amyloid is apparent. The potent binding of AT-02 to amyloid fibrils and heparin is mediated via the multiple electrostatic interactions of the p5R domain; however, absent the peptide, the VH9/VL4 IgG, which exhibits no binding to HS, bound, albeit less avidly, to AL amyloid fibrils (EC_50_ = 74.5 nM). The VH9/VL4 IgG is a humanized variant of the murine 11-1F4 mAb, an antibody generated in our laboratory using a heat-aggregated κ4 light chain as the immunogen. The murine 11-1F4 was shown to bind a cryptic epitope present on misfolded κ and λ light chain variable domains and amyloid fibrils^19,20^. The murine 11-1F4 F(ab)_2_ binds with modest potency to non-κ4 AL amyloid extracts^28^, but this is significantly enhanced by the addition of a polybasic pan-amyloid reactive peptide^28^. Nonetheless, the binding activity of the AT-02 F(ab)_2_ domains may be clinically meaningful, specifically for the treatment of patients with AL amyloidosis.

Following IV administration, AT-02 rapidly bound amyloid in an AA amyloidosis murine model and despite the lability of AT-02 in mouse serum remained detectable in the deposits for more than 7 days following a single injection (Fig. 6). In addition to safety, specificity, and exposure, the amyloid-bound half-life may be a critical factor in determining the therapeutic efficacy of amyloid clearing antibodies. When bound to amyloid fibrils or extract, AT-02 served as an effective opsonin, fixing complement and enhancing FcR-mediated phagocytosis of amyloid extracts by macrophages and monocytes ex vivo (Fig. 5). Importantly, opsonization of amyloid extracts with AT-02 overcame the collagen-mediated ‘don’t eat me’ signal that otherwise prevents effective uptake of amyloid extracts ex vivo^40^.

Therapeutic interventions that slow the progression of amyloidosis, such as protein stabilizers (tafamadis and the recently approved, acoramidis) and mRNA degraders (including vutrisiran and eplontersen) for the treatment of ATTR, or the use of anti-plasma cell immunotherapy (daratumumab) for patients with AL amyloidosis have changed the clinical landscape for patients^8–10^. Amyloid clearance has been observed clinically in some patients with AL amyloidosis who achieve a sustained complete hematologic remission in response to anti-plasma cell therapy, although this is not common^12,41–43^. The mechanisms responsible for autogenous removal of AL amyloid remain unclear, but an innate immune response might be a likely hypothesis^15,44^. Notably, therapeutic stabilizers, silencers, and daratumumab are not designed to directly clear tissue amyloid.

A recent report of three patients with spontaneous regression of cardiac ATTR amyloid demonstrated amyloid reactive polyclonal antibodies in their serum^15^ suggesting that triggering an innate immune response with opsonizing antibodies could be a promising approach for clearing tissue amyloid. Targeted clearance of systemic amyloid deposits and restoration of age-appropriate organ function is a clinically unmet need that might be achieved by a new class of therapeutics – amyloid *depleters* or, perhaps more accurately “*eliminators*”.

Active and passive immunotherapies are not novel approaches to the treatment of amyloid and were pioneered by the use of amyloid-like fibrils as a vaccine in 1999^45^ and with the identification of AL amyloid-reactive antibodies in 2000^46^. Amyloid fibril-reactive antibodies occur naturally in the population and are present in commercial preparations of intravenous immunoglobulin (IvIG)^46^, which has been assessed for the treatment of Alzheimer’s disease (e.g., NCT00818662)^47^. One of the two mAbs being developed as a potential ATTR amyloid depleter, ALXN2220 (NI006), was identified from a pool of naturally occurring antibodies. This human IgG1 binds a linear cryptotope exposed on monomeric TTR but that is less exposed in the native tetrameric form, and as such, the mAb exhibits specific reactivity with nonfibrillar TTR aggregates and amyloid fibrils^48^. ALXN2220 treatment of ATTR amyloid extracts in a murine amyloidoma model resulted in clearance, and in patients, a reduction in cardiac extracellular volume measured by cardiac magnetic resonance imaging (NCT04360434)^49^. The second reagent in development for ATTR clearance, NNC6019 (PRX004), was generated to target a linear peptide sequence that is buried in the interface of tetrameric TTR but exposed on the monomer and in the fibrillar form of the protein^50^. Additionally, two mAbs that are currently being evaluated for AL amyloidosis are cross-reactive IgG. Birtamimab (NEOD001), raised against a linear tetrapeptide from the C-terminal of the amyloid protein A^51,52^, also binds a conserved cryptotope in framework 3 of Ig light chain^53^^,^^54^. The final reagent is the chimeric mAb, anselamimab (CAEL-101; NCT04512235, NCT04504825), which was generated using a heat and acid-denatured κ4 Bence Jones protein. This mAb is proposed to bind a conformational neoepitope formed within the N-terminal 15 amino acids of misfolded light chains and present on both ALκ and ALλ amyloid deposits based on PET/CT imaging of the murine antibody in patients^20,55^.

Relative to any other amyloid depleters in development, AT-02 is a unique reagent. Fusion of the amyloid-reactive peptide enables pan-amyloid reactivity through interactions with two discrete, major components of all amyloid deposits that results in opsonization of the target. This affords an opportunity for clinical evaluation of AT-02 not only in patients with ATTR and AL amyloidosis, but also those with rarer types of the disease, for whom no therapeutic options are available. The preclinical bioactivity data supported the clinical evaluation of AT-02 in patients with amyloidosis (NCT05951049), and motivates additional exploration of the peptide platform for the development of diagnostic^23,56^ and therapeutic strategies^29,57,58^ for amyloidosis.

## Methods

### Antibody production and purification

A humanized IgG1κ VH9/VL4 monoclonal antibody (mAb) was generated from the murine 11-1F4 antibody^19,20,28,59^ by homology modeling and backmutation (Genscript, NJ). Nucleotide sequences encoding a spacer and a peptide (p5R)^26^ were incorporated in-frame at the 3’ end of the VL4 light chain sequence to generate a human IgG1-peptide fusion (AT-02). No FC domain engineering was performed. AT-02 was produced in a perfusion culture of recombinant Chinese Hamster Ovary (CHO) cells under Good Manufacturing Practices (GMP). The AT-02 protein was purified from the perfusion cell culture using standard mAb unit operations, including Protein A and cation exchange chromatography. The integrity of the product was assessed by release testing including electrophoresis and mass spectrometry and was formulated at 50 mg/mL in a buffer containing 20 mM sodium citrate, 140 mM Arginine HCl, and 0.04% (w/v) Polysorbate (PS80), pH 6, (Attralus, San Francisco, CA).

The peptide-free VH9/VL4 mAb control was produced by Genscript or generated in-house following transient transfection of suspension HEK-293 cells and cultured for 7 days. Antibody was isolated from the IgG-depleted cell culture medium by Protein A affinity chromatography. A non-specific human (h)IgG1 antibody was purchased (Sino Biological, PA) to serve as a negative control in binding assays.

### Amyloid-like fibrils, proteins and amyloid

Recombinant λ6 light chain variable domain from patient WIL (rVλ6WIL) was generated in *E. coli* and purified from the periplasmic space^60^. Amyloid-like fibrils of rVλ6WIL were prepared in sterile phosphate-buffered saline (PBS; typically, 137 mM NaCl, 2.7 mM KCl, 10 mM phosphate, [pH 7.2-7.6]) by shaking a 1 mg/mL solution for 72 h at 37°C. Aβ(1-40) peptide was purchased from Anaspec (Fremont, CA) and was treated with trifluoracetic acid (TFA), then hexafluoro-2-propanol (HFIP) to remove aggregates before solubilizing in 2 mM NaOH, then 2x PBS. The Aβ(1-40) solution was centrifuged at 15,000 x g for 5 min to further remove any aggregates that may have formed. The peptide concentration was then determined by HPLC using a standard curve. Fibrils of Aβ(1-40) were generated at 37 °C without shaking, isolated by centrifugation at 15,000 × g for 5 min, and resuspended in PBS. Fibril formation was confirmed by the addition of thioflavin T solution to ~5 μg of fibril preparation and measuring the fluorescence emission at 490 nm (excitation = 450 nm). Human amyloid extracts were prepared from autopsy-derived organs using a modified water floatation method and stored as a lyophilized preparation at RT^61^.

Monoclonal immunoglobulin free light chains (Bence Jones proteins) were isolated from the urine of patients with a diagnosis of AL amyloidosis by preparative zone electrophoresis as described elsewhere^62^. The isolated proteins were characterized by immunofixation and in some cases, amino acid sequence analysis^63^. Peptide p5+14 (GGGYS KAQKA QAKQA KQAQK AQKAQ AKQAK QAQKA QKAQA KQAKQ)^21^, prepared by chemical synthesis and of GMP grade (Ambiopharm), was provided as a lyophilized preparation and stored at -20°C until used (Attralus). Peptide p5+14 with an N-terminal biotin incorporated during chemical synthesis was purchased from Anaspec. The soluble TTR aggregates were generated using a TTR variant (TTRv) with F87M/L110M mutations^64,65^ prone to formation of soluble aggregates under physiologic conditions (2 mg/mL in PBS, for 14 days at 4°C). Soluble aggregates were non-fibrillar as assessed by the addition of thioflavin T and measurement of fluorescence emission at 490 nm.

### Structural evaluation of AT-02

Purified preparations of the AT-02 IgG-peptide fusion and VH9/VL4 antibody were analyzed by gel electrophoresis using 4-12% gradient Bis-Tris polyacrylamide gels (Invitrogen) followed by staining with Coomassie brilliant blue. The proteins were either non-reduced in the sample buffer or reduced by the addition of 5 mM DTT to the sample buffer and boiled for 5 mins before analysis. Size exclusion chromatography was performed using a 7.8 × 300 mm column, 3 mm, 300 Å pore-sized matrix (Agilent Bio SEC-3: 3 mm, 300 Å). A mobile phase of phosphate-buffered saline (PBS) was used with a flow rate of 1 mL/min for 15 min. Samples of 25 μL (~25 μg) were injected, and the eluate was monitored by absorbance at 215 nm.

### Glycan array binding

AT-02 was biotinylated using EZ-Link NHS-biotin kit (Pierce). The binding of biotinylated AT-02 and p5+14 to diverse synthetic glycans was performed by ZBiotech (Aurora, CO). A *n* = 47 glycan array (Supplementary Fig. 2) and *n* = 24 heparan sulfate (HS) array (Supplementary Fig. 3) were probed for the binding of biotinylated AT-02 and biotinylated p5+14 peptide (2 ug/mL), using a background-corrected, fluorophore-streptavidin detection method. The glycans in the array included uncharged hyaluronic acid (HA); heparin, chondroitin sulfate AC (CS-AC), chondroitin sulfate D (CS-D), dermatan sulfate (DS), heparan sulfate with either low-intermediate sulfation or high sulfation, and keratan sulfate (KS).

### In vitro binding studies—Europium-linked immunosorbent assay

Binding potency studies were performed using an europium-linked immunosorbent assay (ELISA). Wells of a 96-well polystyrene microplate (Corning, Corning, NY, USA) were coated by addition of 50 μL of either rVλ6WIL fibrils (0.83 µM), Aβ(1-40) fibrils (0.83 µM), or human amyloid extracts (0.06 mg/mL) and the plates dried overnight at 37 °C. For heparin binding, commercially available heparin-coated plates (Bioworld, Fisher) were used. The wells were incubated with 200 µL of blocking buffer (PBS containing 1% bovine serum albumin; PBSA) for 1 h at RT before washing with PBS. Washing was followed by the addition of the appropriate concentrations of antibody diluted in PBS containing 1% (w/v) BSA and 0.05% (v/v) tween 20. Following another wash step, biotinylated goat anti-human Fc (1:4000 dilution; Jackson Immunoresearch, West Grove, PA) was added. After another PBS wash step, the well was incubated with 100 µL of europium–streptavidin (1;1000 dilution; Perkin Elmer, Waltham, MA, USA) was added for 1 h followed by a PBS wash. Finally, 100 µL of enhancement solution (Perkin Elmer) was added to each well. Time-resolved fluorescence emission was measured using a Wallac Victor 3 plate reader (Perkin Elmer). Means and standard deviations from a minimum of three replicates were plotted (Prism v. 10.1.0), and the concentration of Ab at the midpoint of binding (EC_50_) was calculated using a 4-parameter logistic regression (Prism v. 10.1, Graphpad).

### Surface Plasmon resonance measurement

Surface plasmon resonance was performed using a Reichert 2SPR system (Reichert, Inc. NY, USA). Experiments were performed using either a carboxymethyl dextran chip derivatized with rVλ6WIL fibrils or a streptavidin chip derivatized with biotinylated heparin (Millpore-Sigma, St. Louis, MO). An ethanolamine blocked channel served as the reference control for both chips. Sensorgrams were reported as the difference in resonance units between the rVλ6WIL fibril or heparin channel minus reference channel and the buffer control. Serially diluted samples of AT-02 or VH9/VL4 (starting at 80 µg/mL, equating to approximately 500 nM) were evaluated, and the data collected for >600 s (180 s binding-phase and ~480 s dissociation-phase) with a flow rate of 25 µL/min. On- and off-rate data were extracted from the sensorgram, aligned, and analyzed by fitting to the two-state binding algorithm with conformational change [A + B = AB = AB*], which provided the best fit to the kinetic data based on the χ^2^ statistics (Tracedrawer, Ridgeview Instruments).

### Immunohistochemistry and Histology

Formalin-fixed amyloid-laden or control (amyloid-free heart, liver, spleen, and kidney) tissue sections were deparaffinized and antigen retrieval was performed using a citrate buffer, pH 6 (Dako Corporation, Carpenteria, CA, USA). The tissue sections were then treated with 3% H_2_O_2_ solution for 30 min at RT and followed by washing in PBS and an avidin/biotin block step (Vector Laboratories). Samples were then incubated with AT-02 or the control hIgG1 at 2 µg/mL in PBS overnight at 4 °C. After a wash step, bound antibody was visualized using an avidin–biotin immunoglobulin detection kit (Elite Mouse IgG kit; Vector Laboratories, Burlingame, CA, USA) followed by development with diaminobenzidene (ImmPACT™ Peroxidase Substrate kit; Vector laboratories). Amyloid staining was performed using alkaline Congo red^66^. Brightfield and Congo red fluorescence images were acquired using a Keyence BZ-X700E microscope (Keyence, Atlanta, GA).

### Phagocytosis assays

Synthetic rVλ6WIL fibrils and human amyloid extracts were labeled with the pH-sensitive fluorophore pHrodo Red succinimidyl ester (Invitrogen). Human THP-1 monocytes (ATCC), cultured in DMEM/F-12 (with 1% penicillin/streptomycin, 10% fetal bovine serum, Cytiva, Logan, UT) in the wells of a 24-well tissue culture plate were differentiated into adherent M0 macrophages by overnight incubation in 50 nM phorbol myristate acetate (PMA) followed by a 3-day recovery in the absence of PMA. The culture medium was then replaced with serum-free RPMI (Cytiva), and the pHrodo Red SE-labeled amyloid substrate was added to a final concentration of 20 µg/well and incubated for up to 3 h at 37°C. Phagocytosis of the fibrils or amyloid substrate was quantified by acquiring four non-overlapping fluorescence images per well (Keyence BZ-X700E microscope; Keyence, Atlanta, GA). The number of positive pixels (with intensity above a pre-established threshold) in each image was calculated using Image Pro Premier (Media Cybernetics).

As an alternative to presenting to the cells the amyloid extract in suspension, pHrodo Red SE-labeled fibril and amyloid extracts were coated onto the wells of a collagen-coated 24-well plate by incubation overnight at 37 °C and then exposed to 1 × 10^6^ THP-1 monocytes for 3 h. Any positively stained cells from each well were quantified by spectral flow cytometry (2-laser Cytek Northern Lights, Cytek Biosciences, Fremont, CA). Data was analyzed and visualized using FlowJo ver. 10.8.1 (Becton, Dickinson & Company, Ashland, OR).

### Complement activation

Total complement-activation was assessed by measuring the deposition of active C5b9 in wells containing rVλ6WIL fibrils or amyloid extract in the presence of either AT-02 or hIgG1 in human plasma (1% to 10% v/v). The formation of C5b9 was detected using a biotinylated anti-C5b9 antibody (aE11 clone, AbCam, Cambridge, UK) at a 1:2000 dilution, followed by europium-conjugated streptavidin (Perkin Elmer), as above. Wells without plasma served as additional controls for the non-specific activation of complement.

### Radioiodination

100 µg aliquots of AT-02 IgG-peptide fusion protein or the control hIgG1 mAb were radioiodinated with 2 mCi iodine-125 (^125^I; Perkin Elmer) using 10 µg of Chloramine T. Radiolabeled molecules were then purified by size-exclusion gel filtration using Biogel A1.5 m media (BioRad) with a mobile phase of PBS containing 0.1% (w/v) gelatin. The radiochemical purity and integrity of the products were assessed by SDS-PAGE gel (4-12% Bis-Tris polyacrylamide gels; Invitrogen) using phosphor imaging (Cyclone Storage Phosphor System, Perkin Elmer, Shelton, CT, USA).

### In vivo biodistribution of ^125^I-AT-02 in mice with systemic AA amyloidosis

In vivo biodistribution studies of ^125^I-AT-02 or ^125^I-hIgG1 control were performed to assess specific uptake of ^125^I-AT-02, using small animal imaging, tissue radioactivity measurements and microautoradiography in male and female H2-L^d^-huIL-6 Tg Balb/c mice with systemic AA amyloidosis^67^. Amyloidosis was induced in the mice by intravenous injection of 100 µg amyloid-enhancing factor (AEF; murine AA amyloid extracted from the spleen of an amyloid-laden mouse), and the animals were used within 5 weeks after the induction of amyloid. Mice were administered IV, in the lateral tail vein, ~100 μCi ^125^I-AT-02 or ^125^I-hIgG1 (~10 μg in a 200 µL-volume of sterile PBS containing 0.1% gelatin). Cohorts of mice (*n* = 3) were euthanized at 4 h and 24 h post-injection. Small animal single photon emission and x-ray computed tomographic (SPECT/CT) imaging was performed post-mortem (Inveon Trimodality Platform, Siemens, Knoxville, TN). Thereafter, tissue samples (skeletal muscle, liver, pancreas, spleen, left and right kidney, stomach wall, upper and lower intestines, and heart) were harvested and the tissue radioactivity (expressed as mean percent injected dose per gram of tissue [%ID/g] ± SD) was measured using a gamma counter (Wizard 2, Perkin Elmer). Samples of tissue were also placed in 10% buffered formalin for 24 h for microautoradiographic analyses.

### Microautoradiography

Fixed murine tissues were paraffin-embedded, and 6-μm-thick sections were cut onto Plus microscope slides (Fisher Scientific), dipped in NTB-2 emulsion (Eastman Kodak), stored in the dark, and developed after an approximate 4-day exposure before counter-staining with hematoxylin. Brightfield fluorescence images were acquired using a Keyence BZ-X700E microscope (Keyence, Atlanta, GA).

### In vivo biodistribution of AT-02 in mice with systemic AA amyloidosis

Systemic AA amyloidosis was induced in H2-Ld-huIL-6 Tg Balb/c mice as described above. At approximately 5-week post AEF induction, cohorts of mice (*n* = 3) were intravenously administered 400 μg of AT-02 formulated with approximately 200 μL of sterile PBS. Cohorts of mice were euthanized by isoflurane overdose at 24 h, 72 h, and 168 h post injection of AT-02. The organs were harvested and fixed in 10% buffered formalin for 24 h followed by paraffin embedding and tissue sectioning. The distribution of AT-02 in the liver, spleen, and heart was assessed by immunostaining using an avidin–biotin immunoglobulin detection kit (Elite Mouse IgG kit; Vector Laboratories) followed by development with diaminobenzidene reagent (ImmPACT™ Peroxidase Substrate kit). Amyloid staining was performed using alkaline Congo red. Brightfield images were acquired using a Keyence BZ-X700E microscope (Keyence, Atlanta, GA). Congo red birefringence images were acquired with cross-polarized illumination using a Leica DM500 light microscope equipped with polarizing filters.

### Pharmacokinetics of AT-02 in WT mice

The pharmacokinetics of AT-02 and ^125^I-AT-02 were assessed in WT mice. Mice were administered, via IV bolus injection, 100 μg (~5 mg/kg) of AT-02, with or without doping with ^125^I-AT-02. Between 0.5 h and 168 h post-injection, cohorts of mice (*n* = 3) were anesthetized by isoflurane inhalation, and blood was collected from the retroorbital sinus before euthanasia. The AT-02 content in the serum or whole blood was quantified using a capture ELISA (using a goat anti-human IgG capture reagent and a biotinylated goat anti-human IgG Fc [1:4000 dilution]) or gamma counting for samples containing radioiodinated AT-02. The PK data were evaluated using a non-compartmental model.

### Stability of AT-02 in mouse or human serum

AT-02 was added to mouse serum (CD1 mouse serum, Innovative Research, Sarasota, FL) or freshly prepared human serum at a final concentration of 3 μM. One microliter of AT-02 was added to 99 μL of PBS to serve as a positive control. The samples in mouse serum were incubated at 37 °C for 24 h and in PBS and human serum for 9 days. Aliquots of serum were taken at the time points indicated and frozen for batch evaluation. For bioactivity analysis, the samples were diluted 1:1000 in PBS (to yield a final concentration of 3 nM) and assayed for reactivity using the heparin binding ELISA, as described above. The mean and standard deviation (SD) were calculated from at least three replicates and plotted as the percent of binding relative to a time 0 sample (diluted in each medium but immediately frozen). For display purposes, the data were fit to a single exponential decay model (Prism v. 10.1).

### Colocalization of AT-02 with human AL amyloid in a murine model

AT-02 or control hIgG1 was labeled with the near-infrared fluorophore, NHS-DyLight800 (DL800; Thermo-Pierce, Waltham, MA) in 100 mM sodium bicarbonate buffer, pH 8.3. Unbound dye was removed by dialysis (3000 Mw cutoff) for 24 h against 1 L of PBS, pH 7.2. Localized human AL amyloidomas were generated by subcutaneous injection of 10 mg of human ALλ(CLA) amyloid suspended in 1 mL sterile PBS on the dorsal flank of male and female NU/NU mice. Seven days after the amyloid injection, the mice were administered 700 μg (~200 μL) of DL800-AT-02 or DL800-hIgG1, either intravenously in the lateral tail vein or intraperitoneally (*n* = 5 DL800-AT-02 or *n* = 6 DL800-hIgG1 for each route of injection). Fluorescence images of the mice were acquired under isoflurane anesthesia on days 1, 3, 7, and 10 post-injection (iBox Scientia; Analytik Jena, Upland, CA) using an 800-nm bandpass filter set (2-second exposures with 1 × 1 binning).

### AT-02 mediated phagocytosis of human amyloid in mice

Human ALλ(BAL) extract was labeled with the pH-sensitive dye, pHrodo Red STP ester (Life Technologies)^68^. Prior to injection, the extract was pretreated with 400 μg of AT-02 or PBS vehicle for 1 h. Amyloid extract (2 mg in 0.2 mL), containing 10% (w/w) pHrodo Red-labeled amyloid, was injected subcutaneously (SC) in NU/NU mice (*n* = 5 per group). Mice were treated again, 8 days post-implantation, with 400 μg of AT-02 or PBS vehicle in 0.1 mL, administered SC. Fluorescence images of the mice were acquired under isoflurane anesthesia on days 1, 5, 7, 9, and 12 post-injection (iBox Scientia), using a Cy5 excitation and emission filter set for pHrodo Red (100-milisecond exposure, 1 × 1 binning).

### AT-02 mediated clearance of human amyloid in mice

Human ALλ(CLA) extract was labeled with the near-infrared fluorophore, NHS-DyLight800 (DL800; Thermo-Pierce, Waltham, MA), as described above. Unbound dye was removed by dialysis (as above) with sterile PBS, pH 7.2, after centrifugation at 2000 × g for 15 min. Amyloidomas were generated by SC injection of 2 mg of extract (containing 10% w/w Dylight800-labeled amyloid) in 0.2 mL sterile PBS on the dorsal flank of NU/NU mice (*n* = 5 per AT-02 group and *n* = 4 in the vehicle control group). Prior to injection of labeled extract, the amyloid was pretreated with 400 μg of AT-02 or PBS vehicle for 1 h. At 8 days post-implantation of amyloid material, the amyloidomas were treated again by SC injection of 400 μg of AT-02 (0.1 mL) or PBS vehicle.

Fluorescence images of the mice were acquired under isoflurane anesthesia on days 1, 4, 8, and 15 post injection, (iBox Scientia), using an 800-nm bandpass filter set (2-second exposures with 1 × 1 binning). On day 15, the animals were euthanized by isoflurane overdose, and the residual amyloidomas were exposed, images acquired, and the area occupied by the amyloid was calculated using ImageJ software (http://rsbweb.nih.govij). Residual amyloidomas were then excised, fixed in 10% buffered formalin for 24 h before paraffin-embedding, sectioning, and staining with H&E. Brightfield microscope images were acquired as described above.

### Analysis of optical imaging data

The mean raw density (MRD) of the amyloid-associated fluorescence in the DL800 or pHrodo Red images was quantified by region of interest (ROI) analysis. Freeform ROIs encompassing the amyloid mass were manually drawn on the images by a single reviewer who was blinded to the study design. A single ROI was used for the same animal at each time point. A second ROI covering a representative amyloid-free region of the mouse was drawn and served as the mouse-specific background fluorescence (associated with autofluorescence from skin). The final fluorescence signal intensity was calculated by subtraction of the background MRD from the amyloid-associated MRD fluorescence.

### Statistical methods

Correlation analyses were performed by determining the Pearson correlation coefficient (*r*) using the two-tailed hypotheses. One-way ANOVA analyses were performed with multiple comparison testing using Dunnett’s Correction when significant main effects were detected. Factorial and repeated measures ANOVA analyses were used with a Geisser-Greenhouse correction for sphericity. Multiple comparisons were performed between treatment groups using Šidák’s Correction when a significant main or interaction effect was detected. Outliers were identified using ROUT method with Q = 10%. Comparisons of phagocytosis data were performed using an unpaired student t-test (α = 0.05). In vivo biodistribution data were displayed as the mean and standard deviation (*n* = 3). All analyses were performed using Prism v.10.1 (Graphpad), where: ns, *p* > 0.05; *, *p* ≤ 0.05; **, *p* ≤ 0.01; ***, *p* ≤ 0.001; ****, *p* ≤ 0.0001.

### Ethics

All patient-derived tissue samples were used in accordance with an Institutional Review Board-approved application. Animal studies were approved by the University of Tennessee Institutional Animal Care and Use Committee and were performed in accordance with the guidelines provided by OLAW and the Guide for the Care and Use of Laboratory Animals. The University of Tennessee Medical Center animal program is an AAALAC-i-accredited institution.

## Supplementary information

Supplementary information

## Data Availability

No datasets were generated or analysed during the current study.
