# Supplementary material for: The antibody-peptide fusion protein, AT-02, is an effective opsonin with pan-amyloid reactivity
Source: NPJ Drug Discov. 2025 Aug 4;2:18. doi: 10.1038/s44386-025-00015-4 (PMC13267120; doi:10.1038/s44386-025-00015-4)

Supplementary Information

Mass spectrometric analysis of AT-02

Intact reduced and deglycosylated mass analyses were performed on the AT-02 protein using liquid chromatography-coupled mass spectrometry (LC-MS) using a Xevo G2 (Waters) time-of-flight (Q-TOF) MS system coupled to an Acquity (Waters) ultra-high performance liquid chromatography (UHPLC) system with a PLRP-S 1000 Å column (Agilent). Data were processed using Protein Metrics Inc. (PMI)-Suite/Intact MS software.

AT-02 samples were diluted and the N-linked glycans were removed by treatment with PNGase F. The deglycosylated protein was then reduced by the addition of dithiothreitol (DTT) under non-denaturing conditions. Based on these conditions, it is likely that only inter-chain disulfide bonds were reduced while intra-chain disulfide bonds remained intact, resulting in a partially reduced molecule.

Supplemental Figure 1: Intact mass measurements of reduced and deglycosylated AT-02 light and heavy chains.

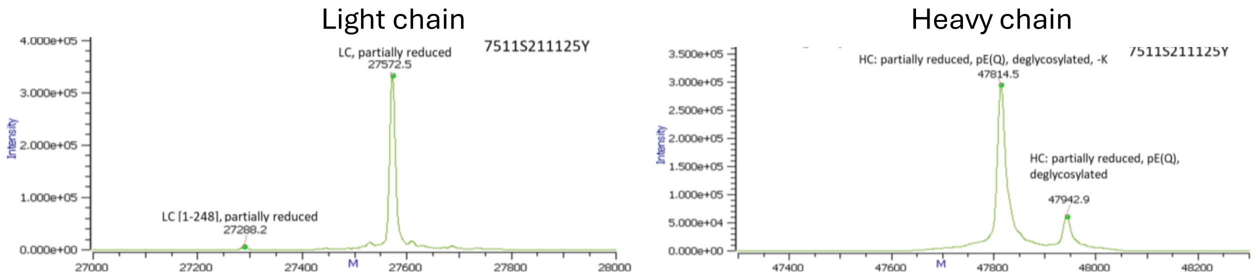

Supplemental Figure 2: Glycosaminoglycan (GAG) array structures shown in SNFG format

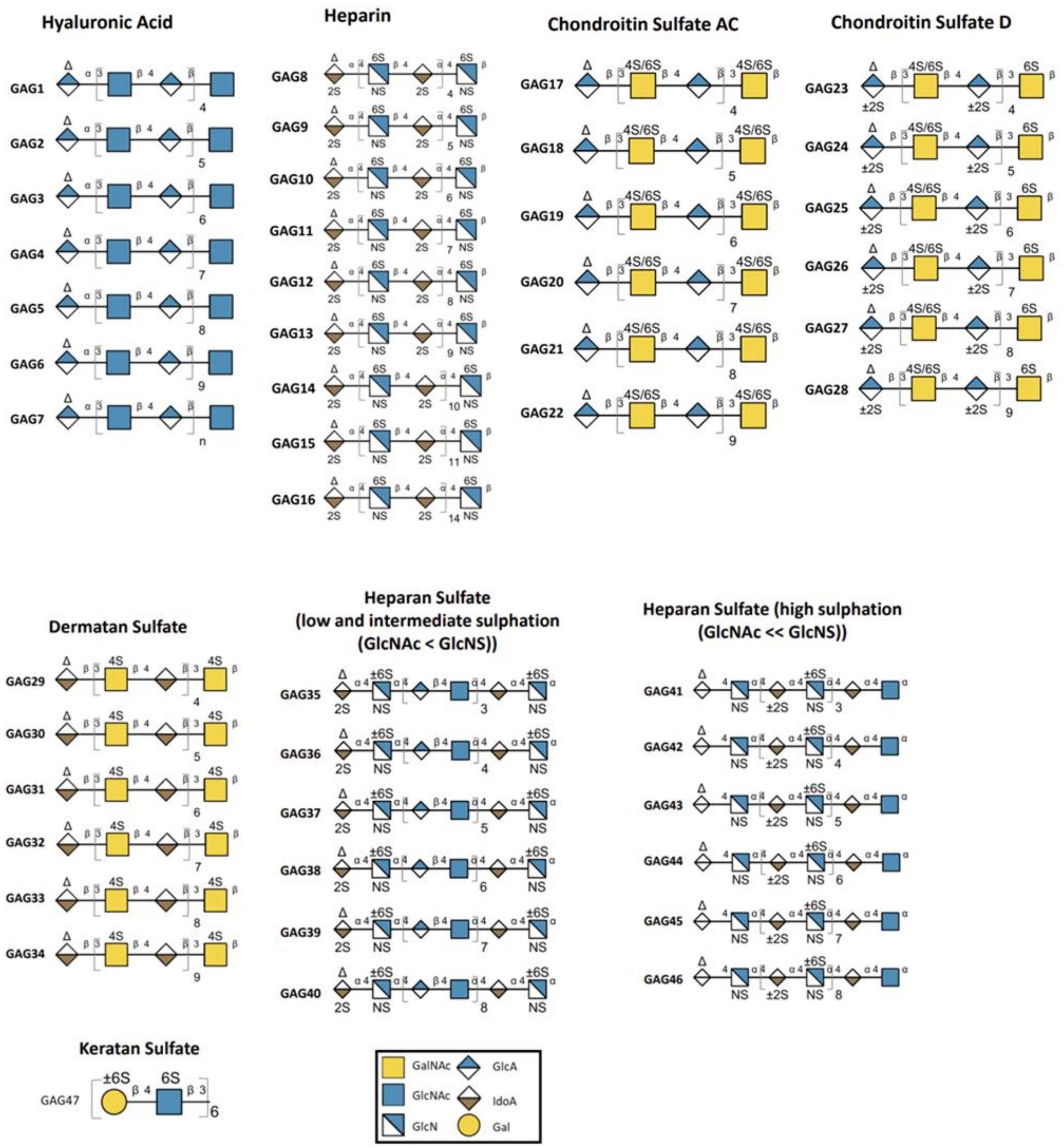

Supplemental Figure 3: Heparan sulfate (HS) array structures shown in SNFG format

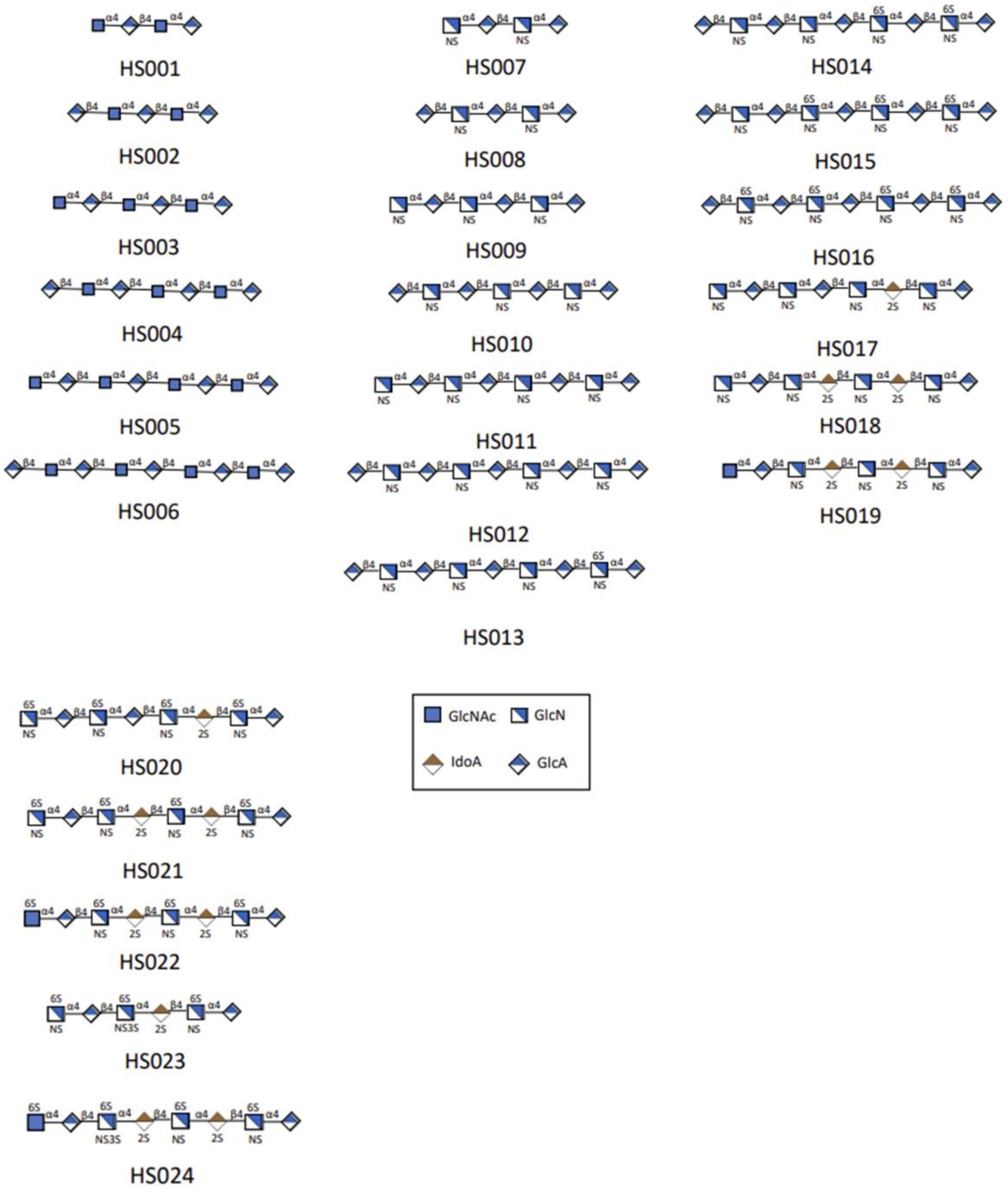

Gel electrophoresis Supplementary Information

Uncropped NuPage® Novex® 4-12% Bis-Tris Gel with MES used to generate Figure 1B. All lanes came from the same gel (as shown below) but were cropped to remove blank lanes and re-ordered to generate Fig 1B.

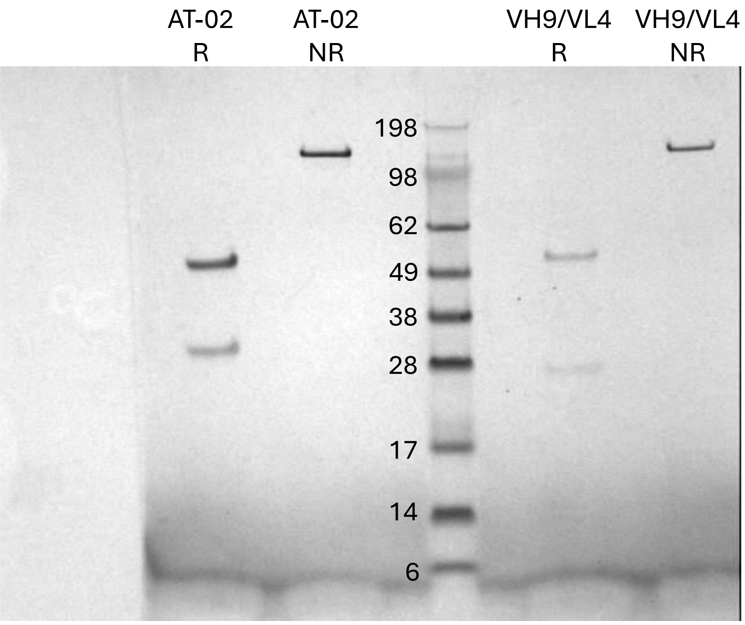

SeeBlue® :Plus2 Pre-Stained Standard with apparent molecular weight on a NuPage® Novex® 4-12% Bis-Tris Gel with MES, as used in this study.

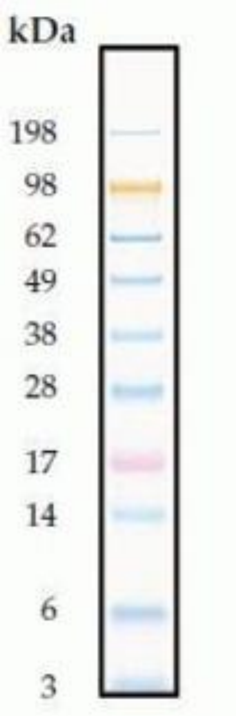

Supplement: Supplementary file 1 — Supplementary information [file 44386_2025_15_MOESM1_ESM.pdf]
